# Editorial: Catalysis in Iberoamerica: Recent Trends

**DOI:** 10.3389/fchem.2022.870084

**Published:** 2022-03-08

**Authors:** Andrea Alvarez Moreno, Pedro Arcelus-Arrillaga, Svetlana Ivanova, Tomas Ramirez Reina

**Affiliations:** ^1^ Estado Sólido y Catálisis Ambiental, Departamento de Química, Facultad de Ciencias, Universidad Nacional de Colombia, Ciudad Universitaria, Bogotá, Colombia; ^2^ Department of Chemical Engineering, Faculty of Engineering and Informatics, University of Bradford, Bradford, United Kingdom; ^3^ Centro Mixto Universidad de Sevilla-CSIC, Instituto de Ciencia de Materiales de Sevilla, Sevilla, Spain; ^4^ Department of Chemical and Process Engineering, University of Surrey, Guildford, United Kingdom

**Keywords:** catalysis in Iberoamerica, trends in catalysis, Iberoamerican scientific community, catalysis for energy, catalysis for environment, heterogeneous catalysis, reaction engineering

**FIGURE 1:**
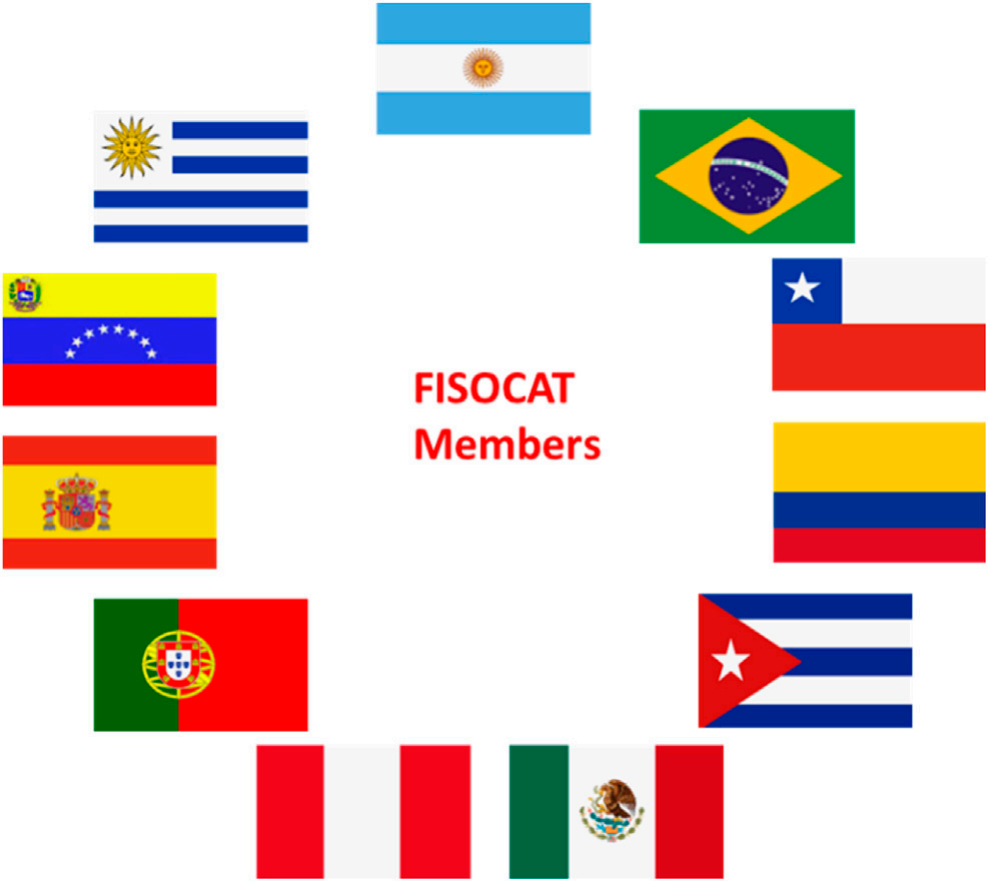
FISOCAT members.

One of the most dynamic fields in contemporary chemistry and engineering is catalysis, which is essential for multiple industries and the world’s economy. The field of catalysis started to develop in Latin America 40 years ago in relation to the needs of emerging oil industries and technological infrastructures. Since then, numerous scientific collaborations have been established between different countries giving birth to the Iberoamerican Catalytic Society represented currently by the Iberoamerican Federation of Catalysis Societies (FISOCAT, from Spanish Federación Iberoamericana de Sociedades de Catálisis). FISOCAT is a not-for-profit organization, integrated by 11 member countries (Figure 1), whose objective is to stimulate scientific and technical interchange between the Catalysis Society members.

The scientific relationship between member countries is also reflected in the organization of a biannual scientific meeting “Congreso Iberoamericano de Catalisis” that started in 1968 in Spain with 51 participants. The meeting has since grown in relevance and in participation to gather more than 400 participants in the last two meetings celebrated in Portugal in 2018 and Mexico in 2020 (Gomes and Faria, 2020; Vessuri, 2020). The formation of FISOCAT and the continuous increase of participants in catalysis societies and meetings is a clear indication of the successful establishment of academic and scientific exchange and collaborations allowing Iberoamerican research to be confirmed as a hotspot for the international catalytic field.

This research topic gives proof not only of the vast scientific exchange between different catalysis groups, but also that Iberoamerican researchers are at the forefront of catalysis research. Contributions addressing major fundamental and industrial challenges have been included, showing the great expertise and enthusiasm of Iberoamerican researchers working in modern fields such as biogas conversion, CO_2_ and biomass valorization, fine chemistry, and photocatalysis.

This research topic contains an excellent review by Sánchez-López et al. (Sánchez-López et al., 2021) presenting some recent advances in catalysis based on transition metals supported on zeolites. The review focuses on various challenging issues such as CO_2_ capture and conversion, methane to methanol transformation, H_2_ storage, and biomass and polymeric waste upgrading.

CO_2_ and CH_4_/CO_2_ conversion are the main subjects of three more contributions presented by Gandara-Loe et al. (Gandara-Loe et al., 2021), Alvarez Moreno et al. (Álvarez Moreno et al., 2021), and Le Saché et al. (le Saché et al., 2021). While the first highlights some important advances in reverse water gas shift reaction, the other two are focused on dry reforming of methane. All three contributions used nickel-based catalysts clearly showing the versatility of this metal as the active phase in CO_2_-involved reactions for syngas production. Nickel can be used either as a monometallic substance or alongside La, Zr, K, or Ru, and these metals are charged to increase active phase stability and overall catalyst durability.

In this research topic, biomass conversion is studied by Martin Kessler and Roberto Rinaldi (Kessler and Rinaldi, 2022) via mechanocatalytic depolymerization of lignocellulose. The authors propose energy dose as a unified metric for mechanocatalytic depolymerization result comparison. In such an approach, the trial-and-error strategy can be rationalized with the premise of saving valuable time in experiments assisted by mechanochemistry.

The isomerization of allyl alcohol over Ru-based catalysts is studied by Enciso et al. (Enciso et al., 2021). In this work, a mechanism involving allyl alcohol adsorption and migration to Lewis acid sites available on the metal/support interface with the formation of Ru-alcoholate and β-hydride elimination and n-propanal formation is proposed.

The implementation of carbon-based catalysis is also reflected in this research topic. Shen et al. (Shen et al., 2021) studied carbon dots doped with TiO_2_ sheets for photocatalytic pollutant degradation. The hybrid materials, designed to increase the photocatalytic efficiency under visible light, showed that the addition of carbon dots extended the light absorption and diminished the rate of recombination by promoting charge transfer. In another contribution by Soares et al. (Soares et al., 2021), an activated carbon-supported Pt-In catalyst is implemented for the reduction of nitrites in water. Nitrogen selectivity was found to increase with indium content due to Pt-In alloy formation reflecting in different electronic properties of the active surface and thereby higher reduction efficiency.

As has been mentioned in this editorial, the spirit of the research topic is to highlight the relevance of the work done by the Iberoamerican scientific community in the field of catalysis. The eight manuscripts included offer great representation of the quality and relevance of the contributions of Iberoamerican scientists and research groups to address the greatest challenges society and industry are facing as well as to contribute toward the development of the catalysis field. The topic editors expect that this research topic will serve as a representation of the work and collaborations in the area of catalysis developed in Iberoamerica, and to promote its growth and impact worldwide.
